# ‘I’m not an anti-vaxer!’—vaccine hesitancy among physicians: a qualitative study

**DOI:** 10.1093/eurpub/ckab174

**Published:** 2021-09-28

**Authors:** Franziska Ecker, Ruth Kutalek

**Affiliations:** Department of Social and Preventive Medicine, Center for Public Health, Medical University of Vienna, Vienna, Austria

## Abstract

**Background:**

Over the last years, research interest in vaccine hesitancy has increased. Studies usually focus on perceptions of parents and have largely neglected the group of health care providers. However, doctors’ notions on vaccination have a major impact on the decision-making process of their patients. We were interested to understand the phenomenon of vaccine hesitancy among physicians, with a particular focus on the measles vaccine. Furthermore, we aimed to understand the underlying perceptions of measles that may be associated with vaccine hesitant decisions.

**Methods:**

In order to get an in-depth view, semi-structured interviews with physicians were conducted. Doctors were eligible for the study if they articulated vaccine hesitant views and/or demonstrated vaccine hesitancy in their medical practice.

**Results:**

We interviewed 12 physicians, of whom 11 had a medical practice with no contract with the Austrian social insurance (‘Wahlarzt’) and additional training in complementary and alternative medicine. We found perceptions of immunology, health and illness that were discordant with evidence-based medicine and closely related to alternative and complementary medicine. All participants argued for a delayed administration of the measles vaccine. We found a consistent inclination towards ‘individual vaccination’, which was explained as empowering parents and to strengthen their decision-making competencies. Most participants expressed doubts about the reliability of vaccine studies and were concerned with possible long-term effects.

**Conclusions:**

Paying closer attention to doctors’ concerns on vaccination might help to design target-oriented interventions to specifically strengthen vaccine confidence.

## Introduction

In 2019, global measles cases reached an all-time high of the last quarter-century. Between 2016 and 2019, global annual case numbers climbed up from 132 325 to 869 770. Austria reported 77 cases in 2018 and 151 cases in 2019, hence, it ranks in the midrange of the EU.^1–3^ Measles is a highly contagious viral disease. Stable vaccination coverage rates of ≥95% with two doses of a measles containing vaccine (MCV) are therefore crucial, as only a slight reduction in coverage would result in multiple times increased case numbers.^4^^,^^5^ Vaccine hesitancy (VH), defined by the SAGE working group as ‘delay in acceptance or refusal of vaccines despite availability of vaccine services’,^6^ is of growing scientific interest and has the potential to undermine measles immunization rates.^7^ The term VH comprises a broad spectrum of attitudes and beliefs, associated with different vaccination behaviour and increased request for alternative vaccination schedules.^8^

Research on VH has mainly focussed on parents.^9^^,^^10^ Among them, key elements of VH include risk conceptualization (e.g. the weighting of the perceived risk of a vaccine vs. the disease), alternative health beliefs (e.g. ‘the vaccine is not natural’ and ‘children’s bodies are overcharged by vaccines’), philosophical considerations on parents’ responsibility (e.g. parents want to take self-determined health decisions for their children and they do not want to be pushed towards a certain decision) and distrust towards the pharmaceutical industry, public health authorities and health providers (e.g. these institutions only have financial interests and health providers are influenced by them).^10^^,^^11^

There is no data available on the quantity of VH among Austrian doctors. Studies from other European countries, however, showed that the vast majority of medical doctors is favourable towards vaccination, and that there is a small percentage who is sceptical.^12–15^ The use of complementary and alternative medicine (CAM) is regarded as a possible factor for VH.^16^^,^^17^ According to Bean et al.^18^ common beliefs among CAM practicing doctors included that ‘the body is able to defend and heal itself’, ‘disease is an expression of poor personal management’ and ‘the children’s immune system is too weak to bear a vaccine’. Deml et al.^19^ found that the basis of VH among CAM practicing doctors is the firm desire to acknowledge each person’s individuality and the will to empower parents to make self-determined decisions.

The Austrian social insurance is based on the principle of solidarity. Everyone covered by social insurance (in 2017 99.9% of all inhabitants^20^) has access to good quality medical care. Doctors who have a contract with the social insurance (‘Kassenarzt’) settle the costs directly with them and patients do not have to pay. Doctors who do not have a contract with the social insurance are called ‘Wahlarzt’. Patients have to pay these services and get it partly refunded by the social insurance. In contrast to a ‘Kassenarzt’, a ‘Wahlarzt’ is able to decide freely on opening hours, the amount of patients as well as the provided services and its costs.^21^^,^^22^

According to Kemppainen et al.^23^ around 36% of the Austrian population used at least one type of CAM in a period of 1 year. This was the third highest percentage found in the EU (after Switzerland and Germany). The Austrian public health insurance does not refund CAM products or interventions.^24^

Vaccination is not mandatory in Austria. Thus, it is essential that health care providers have an affirmative notion concerning vaccination, since their opinions have a strong influence on patients.^25^^,^^26^ An in-depth understanding of VH among doctors is crucial in order to implement target-oriented interventions to counter this growing challenge. It was therefore the aim of this study to investigate beliefs and characteristics of vaccine hesitant doctors concerning (i) vaccination in generally, (ii) specifically the measles vaccine and (iii) their perceptions on measles.

## Methods

Participants were eligible for the study if they (i) practiced as general practitioners (GPs) or paediatricians (Ps), (ii) were by definition ‘vaccine hesitant’ and (iii) provided vaccination information or advice, and/or vaccination services.

We anticipated that it would be challenging to approach vaccine hesitant doctors, as they are considered a minority (e.g. Reference^15^) Furthermore, VH is broadly debated in Austria^27^ and several anti-vaccine doctors who articulated their beliefs in public recently lost their licence as physicians (e.g. Reference^28^). Three different sampling approaches were applied:^29^ (i) F.E. enquired the contact details of two vaccine hesitant doctors through personal contacts (convenience sampling). (ii) Doctors who appeared to be vaccine hesitant judging from their web-page-content were approached (purposeful sampling). (iii) Through recommendations of previous participants, we were able to contact further potential participants (snowball sampling). Physicians were contacted via telephone to explore whether the inclusion criteria were met. In total, we contacted 20 doctors. Three doctors never reacted to the interview request and five doctors refused to participate in the study.

Participants were asked to indicate demographic data, such as age, sex, specialization (general medicine or paediatrics; additional training in CAM) and type of practice (‘Kassenarzt’ or ‘Wahlarzt’). An interview guide with open-ended questions was prepared that mainly covered the following topics: perceptions on measles (risks, complications, treatment and experience), opinion on vaccination (early childhood vaccination and polyvalent vaccines) and especially on the MCV (timing, side effects and necessity). Data collection and analysis were performed after the participants’ informed consent was obtained. The interviews were held in German, lasted between 40 and 150 min and were conducted in the doctors’ practices. During the process of verbatim transcription, interview data were anonymized by using one of the 12 most frequent last names in Austria as a pseudonym. In qualitative research, the interpretation of data are not considered independent from sampling and further sampling takes place after the interpretation of earlier data.^29^^,^^30^ We conducted an inductive content analysis, applying techniques of grounded theory:^29^^,^^31^ (i) open coding in order to reflect the text and identify categories; (ii) axial coding, in which the codes are broken down, modified and compared until relationships and variations are understood; and (iii) selective coding to determine core categories. During the research process analytic memos were made whenever new insights were gained. The coding was conducted with atlas.ti (version 8.4.20.0).^32^ As is typical for qualitative research,^29^^,^^33^ the sample size was not determined in advance but continued until theoretical saturation was reached, which depended on the informative value and variety of participants. Theoretical saturation is reached when no new or relevant information emerges from additional interviews.

To ensure quality and credibility of data, authors met on a regular basis in order to discuss the findings and to reach consensus. Furthermore, all analytic decisions were documented in detail to ensure consensus.

## Results

Between May 2019 and January 2020, we conducted 12 semi-structured interviews with 6 female and 6 male participants aged between 42 and 73 years. Seven participants were GPs and five were Ps. Eleven doctors worked as ‘Wahlarzt’ and had further training in CAM, eight practiced homeopathy (HP). Only one doctor was a ‘Kassenarzt’ and did not have further training in CAM (Table 1).

**Table 1:** Demographic data

Pseudonym	Gender	Age	Specialization	CAM-type	Type of practice
Dr Wagner	♀	71	GP	Homeopathy	Wahlarzt
Dr Gruber	♂	64	Paediatrician	Homeopathy	Wahlarzt
Dr Winkler	♂	52	GP	Homeopathy	Wahlarzt
Dr Weber	♂	65	GP	Other	Wahlarzt
Dr Huber	♀	46	Paediatrician	Homeopathy	Wahlarzt
Dr Bauer	♀	42	Paediatrician	Anthroposophic medicine	Wahlarzt
Dr Wimmer	♀	51	GP	Homeopathy	Wahlarzt
Dr Müller	♂	73	Paediatrician	Anthroposophic medicine	Wahlarzt
Dr Wallner	♂	59	GP	—	Kassenarzt
Dr Wolf	♀	43	Paediatrician	Homeopathy	Wahlarzt
Dr Stein	♂	42	GP	Homeopathy	Wahlarzt
Dr Pichler	♀	57	GP	Homeopathy	Wahlarzt

Participants’ demographic data characterized by pseudonym, gender, age, specialization, CAM-type and type of practice (private or panel).

GP, general practitioner; P, paediatrics; HP, homeopathy; AnM, anthroposophic medicine.

In the following sections, we will firstly describe the participants’ perception on childhood diseases in general and on measles specifically, and on vaccination. Secondly, we will present their particular positions concerning the measles vaccine. Thirdly, we will explain the participants’ preference for selective vaccination and its implications. Finally, we will describe issues of trust towards the medical system (Table 2).

**Table 2:** Main findings

**Perception of measles and other childhood diseases**	
‘Measles is a manageable childhood disease’	
‘Measles and its risks are often depicted in an exaggerated way’	
‘Childhood diseases have positive aspects’	
‘Childhood diseases can be accompanied with CAM measures’	
‘Childhood diseases should not be suppressed’	
**Perception of vaccination**	
‘Unvaccinated persons are healthier’	
‘Vaccination impairs the neurologic development’	
‘Vaccination impairs the immunologic development’	
‘Combined vaccines overcharge a young organism’	
‘Vaccination at a young age should be avoided’	
**Approaches concerning the measles vaccine**	
‘Vaccines are better tolerated when a child is older’	→ Delay of the MCV, but before kindergarten
‘Vaccines are more effective when a child is older’	
‘Measles implicates a high risk’	
‘Measles infection has positive aspects’	→ Delay of the MCV until puberty or rejection of the MCV
‘Measles infection is manageable’	
‘MCV implicates a high risk’	
‘Monovalent measles vaccine can be considered’	
**Individual vaccination**	
‘Every child is different’; decisions only at an individual level; vaccination is an option	
‘Parents should make self determined decisions’ (gathering information + intuitive decision)	
‘Individual vaccination is not for everybody’ (high effort and costs)	
‘Vaccination is for lower socioeconomic classes’	
**Mistrust against public health authorities and pharmaceutical companies**	
‘Studies often have a conflict of interest’	
‘Long-term effects, soft side effects and interactions are not sufficiently investigated’	
‘Public vaccination schedules are not evidence-based’	
**Criticism of the medical system**	
‘Patients want to see a “Wahlarzt”, as they were disappointed by a “Kassenarzt”’	
‘A “Kassenarzt” does not have time and resources for information seeking parents/patients’	

Summary of the main findings structured in six sections: (i) perception of measles and other childhood diseases; (ii) perception of vaccination; (iii) approaches concerning the measles containing vaccine; (iv) individual vaccination; (v) mistrust against public health authorities and pharmaceutical companies; and (vi) criticism of the medical system.

### Perception on childhood diseases, measles and vaccination

Several participants did not find it necessary to prevent measles. Dr Gruber [♂, 64 years, paediatrician (P), homeopathy (HP)] is a case in point by explaining that, ‘measles is definitely manageable, if a patient is infected at a certain age’. This ‘preferable age’ of being infected with measles (i.e. because of ‘low rates of complications’) lies, according to some participants, between 1.5 and 9 years. It was often highlighted that in their own childhood measles was an ordinary childhood disease, thus, they did not necessarily see the need for preventing it. Some participants reported memories of their own measles infection, in which relational aspects were highlighted. The disease was described as an ‘intensive time’, closely spent with a family member. In this context, some participants criticized modern family structures and gender roles and they blamed vaccination for being a tool to compensate a lack of care for children. Dr Gruber continued: ‘Families are not equipped anymore to care for a highly febrile patient for two or three weeks. Mother is working, father as well and grandmother is at the golf course. That’s how it is. […] If families have a great structure and the grandmother is available for the child, it’s okay. Why shouldn’t this child get measles?’.

It was assumed that most measles complications could be avoided if handled in the appropriate way. Participants believed that measles patients were often hospitalized due to a lack of confidence and experience among young doctors, which in turn created a wrong image of measles. It was emphasized that it was crucial to never reduce fever by pharmaceutical means and to order strict bed rest. In addition, CAM treatments or measures were frequently brought up (e.g. HP, enemas and dietetic measures). Dr Müller (♂, 73 years, P, anthroposophic medicine), who had a very figurative notion of health and illness, explained that ‘measles is a highly febrile illness and it’s very important that children learn in advance, in terms of other febrile infections, how to deal with fever’.

Some participants pointed out that childhood diseases do have beneficial aspects, e.g. for the immune system or the neurologic development of the child, others viewed it as a ‘natural milestone’ in a child’s development. Subsequently, Dr Müller continued that a childhood disease is ‘the first thing a child can do on its own’ and therefore ‘represents the independent will of a child’. Dr Wagner (♀; 71 years, GP, HP) who refused all vaccines claimed that childhood diseases are ‘cleaning up the body’ and are there ‘to get rid of hereditary things’.

It was a common belief that one should not interfere with nature, as every illness had its purpose and should therefore not be ‘suppressed’ by a vaccine. Several participants assumed that the eradication of diseases is neither constructive, nor possible. For example, Dr Pichler (♀, 57 years, GP, HP) reckoned that ‘if you eradicate a disease, another will appear’ and that this phenomenon is ‘obviously a principle of nature’.

It was commonly expressed that unvaccinated persons are overall healthier than vaccinated ones. Dr Wagner was sure that there is a ‘difference in the development’ and she experienced that ‘vaccinated children get chronic diseases, while unvaccinated children enjoy their healthy life’. According to some participants, especially vaccination at an early age has the potential to interfere with the natural development of (i) the immune system and (ii) the neurologic system. Consequently, vaccination would lead to (i) allergies and autoimmune diseases and (ii) to neurologic disorders (e.g. behaviour change, language deficits, loss of trust and happiness). Dr Winkler (♂, 52 years, GP, HP) speaks for the majority of participants when he stated: ‘Vaccines are better tolerated when a child is older! The amount of vaccines infants receive is insane. I believe that these are too many toxins for a small organism’. In line with Dr Winkler, most participants were concerned with polyvalent vaccines, which would ‘overcharge the young organism’, whereas Dr Wimmer (♀, 51 years, GP, HP) brought up: ‘I sometimes think if I wait some time, I tend to prefer combined vaccines, […] as the more single vaccines are used, the more additives are injected’.

### Particular notions on the measles vaccine

No participant complied with the guidelines of the Austrian vaccination schedule regarding the MCV. Dr Wallner (♂, 59 years, GP, no CAM-training, ‘Kassenarzt’) was the only participant who indicated that a specified age for the MCV was necessary (in his opinion MCV1 with 14 months, MCV2 4 months later) and who considered it highly necessary to prevent measles in any case.

Concerning the measles vaccination strategy of the other 11 participants, 3 main approaches emerged: (i) delayed administration of the MCV, but before kindergarten entry, (ii) a delay of the MCV until puberty (the time when the risk of complications increase) or (iii) complete rejection of the MCV. Supporters of the first approach aimed to protect children from getting measles, but preferred the delay of the vaccine due to a supposed enhancement of immune response and a decrease in side effects. Supporters of the second and third approach pursued the target of acquiring the infection due to perceived benefits of the disease. Dr Huber (♀, 46 years, P, HP) was ‘not convinced that the shots should be given at nine months’, as studies would indicate that ‘the earlier children get vaccinated, the less antibody levels rise’. In the same manner, several participants criticized that early vaccination would lead to weak immunity levels among the whole population and therefore to an impaired transferral of maternal antibodies to newborns. This would result in decreased nest protection of infants and to an increased susceptibility of adults, who lose their initially weak protection. Dr Bauer (♀, 42 years, P, anthroposophic medicine) who shared this view explained that for that reason she was ‘not sure if it was a right public health decision to introduce the measles vaccine’*.* Dr Wolf (♀, 43 years, P, HP) also applied this approach to her own children: ‘Well, I just took the chances and let as much time as possible pass by to give them the chance to develop a natural immunity by catching their diseases. And if they do not catch a disease till adolescence, I vaccinate them’.

Some participants condoned great effort and costs for their patients by ordering monovalent measles vaccines from other countries, as they are not available in Austria. These interviewees usually argued that there is a lack of necessity to vaccinate girls against mumps and boys against rubella.

Even though participants named minor and even severe side effects of the MCV, they were not considered as major cause for their doubts. However, participants often expressed a perceived lack of studies investigating the safety of vaccines (see chapter Criticism on biomedicine and the current medical system).

### Individual vaccination—rejecting the ‘one size fits all’ model

Eight participants had an ambivalent point of view towards vaccination and repeatedly used the expression ‘individual vaccination’. Dr Stein (♂, 42 years, GP, HP) emphasized: ‘I am not an anti-vaxer, I am a supporter of individual vaccination!’, while Dr Wolf (♀, 44 years, P, HP) proposed to use vaccines in the same selective manner as antibiotics and stated: ‘I do not call vaccines bad or good. Similarly to antibiotics, I only use it if it’s necessary’. Another frequently addressed aspect that influenced the decision for or against a vaccine was the socioeconomic background of a family, which is exemplified by Dr Weber’s (♂, 65 years, GP, other CAM specialization) statement: ‘My opinion is that vaccination is an option, which is definitely beneficial for certain social classes, but not necessarily for everybody. […] Not vaccination for everybody, but vaccination for risk groups!’ Furthermore, participants raised the dilemma that healthy and stable children tolerate vaccines, but do not need it, whereas weak children could neither manage the disease, nor the vaccine.

Participants indicated to support self-determined decision of parents concerning the selection of vaccines for their children. This approach included two main aspects: (i) the support of the parents’ information seeking process and (ii) the encouragement of the parents’ personal intuition and feeling towards vaccines, which was considered as important tool to choose the vaccines or to decide against vaccination. For that reason, ‘neutral’ or ‘objective’ information was provided by the doctors, book or web-page recommendations were given and intensive discussions were held. Consequently, ‘individual vaccination’ was described as an active involvement in the child’s health, contrary to the passive way of using the offered public vaccination schedule. Hence, the interviewed doctors frequently repeated that no recommendations on the vaccination procedure were given, as they did not want to influence or push parents towards a certain decision. Dr Wolf (♀, 44 years, P, HP) explained: ‘Individual consideration is as important as herd immunity. My choice for an infection is at least well-considered’.

Some participants highlighted that ‘individual vaccination’ is not the adequate approach for everyone. Dr Pichler (♀, 57 years, GP, HP) stated that ‘the Austrian vaccination plan is completely reasonable and should be followed by the biggest part of the population’. She concluded that ‘individual vaccination is only possible for educated people who reflect about the topic and actively decide to spend money to consult a “Wahlarzt”’. Similarly, Dr Wolf explained, ‘It is definitely a two-class medical system, which is determined whether someone has time and money to visit a ‘Wahlarzt’. […] But I think a lot of people do not want or need vaccination advice anyway’, and she concluded that these persons should stick to the vaccination schedule.

### Criticism of biomedicine and the current medical system

All participants distrusted formal medical science, classical biomedicine, public health authorities and the pharmaceutical industry. It was pointed out that vaccine studies had several deficits, predominantly the insufficient investigation of interactions between vaccines, long-term side effects and ‘soft side effects’ (e.g. loss of trust and happiness, slightly increased susceptibility for other infections). Most participants assumed that the pharmaceutical industry influences public health decisions concerning vaccination and that these decisions are often not evidence based. For example, Dr Stein (♂, 42 years, GP, HP) was sure that the Austrian vaccination schedule was not evidence based and emphasized: ‘Vaccination is a highly dogmatic topic among the scientific community and this is something that is insanely difficult to break down with evidence’.

Participants criticized biomedically oriented ‘Kassenärzte’ and indicated that parents increasingly demanded the help of ‘Wahlärzte’ and especially of those who practice CAM, as classical doctors (i) have limited time, (ii) send patients away who ask critical questions, (iii) reject patients who report side effects, (iv) dismiss patients, if they do not accept the vaccination schedule or (v) do not know how to treat side effects of vaccines. In this context, the participants reported several narratives, e.g. ‘During a mother-child examination, the doctor came with the shot and without saying anything he vaccinated the child. The mother didn’t want that! Well, I can understand that!’ (Dr Wallner, ♂, 59 years, GP, no CAM-specialization, ‘Kassenarzt’).

## Discussion

This is the first qualitative study investigating beliefs and opinions among vaccine hesitant doctors in Austria. We could show a relation between CAM and VH as well as between VH and elitism. We describe the notion of ‘individual vaccination’ and the accompanying preference to encourage intuition-based self-determined vaccination decisions among parents. Participants largely distrusted the medical system and questioned the role of evidence-based medicine. Notions specifically related to the MCV were rare, as their criticism rather referred to vaccination in general.

### Illness is not an enemy or the call for ‘individual vaccination’

Participants’ perceptions on health, illness and the immune system were often not in line with evidence-based medicine and underline the relation of VH and CAM.^17^^,^^34^ Participants did not want to be considered as ‘anti-vaxxers’. They argued to make selective use of vaccines and weighed every single vaccine for each patient. These findings are in line with Wardle et al.^19^ and Deml et al.,^21^ who called the pro- vs. anti-vaccination dichotomy into question and supported a more nuanced view of the phenomenon of VH.

To understand these findings it is important to be aware of the underlying concepts of CAM, especially HP. HP presumes that an inner energy determines the level of health, whereas illness is perceived as ‘something from outside’ that causes disturbance of the body’s energy, which is called ‘the vital forces’. In contrast to biomedicine, HP views symptoms as visible effort of the ‘vital forces’ to rebuild health, and thus, as something positive.^35^^,^^36^ Several participants believed in an innate intelligence of nature and the body (see Results: Perception on childhood diseases, measles and vaccination), which relates to the concept of ‘vital forces’, whereas non-natural measures (often called ‘external measures’ by our participants, i.e. vaccines) are considered an interruption of the innate intelligence. This may be illustrated by the participants’ high trust in the children’s immune system and its ability to fight childhood diseases, while insisting that young children are too weak to bear a vaccine (see Bean et al.^18^) ‘Why shouldn’t a child get measles?’ was a central question raised by many participants. In a similar finding by Deml et al.,^19^ participants used the expression ‘the right to be sick!’, underlying the belief in beneficial aspects of measles.

In our study participants argued that ‘individual vaccination’ was not for everyone, especially not for lower social classes or uneducated persons, as it needed a high level of commitment and knowledge. Similar notions were also found in other studies among vaccine hesitant parents.^10^ An Australian study revealed that highly educated parents believed in their ability to control the health of their children without vaccines, since their general success enables them to control their lives.^37^ Consequently, our results underline that VH is associated with healthism, elitism and the belief to stand out of the crowd.

### Patient-centeredness or the frustration with the Austrian medical system

Participants frequently indicated that the most important factor to determine the individual choice of vaccines is the parents’ intuition. These findings are similar to Attwell et al.^38^ who described a ‘do-it-yourself’ attitude in terms of vaccination decisions that was promoted and supported by CAM providers. Some participants had difficulties to specify their preferred vaccination strategy (timing, quantity, monovalent vs. polyvalent, rejection), as no general concept exists in their point of view. Others who indicated a preferred strategy highlighted that every individual decision-making had to be acknowledged even if families did not concur with the physician’s opinion. These findings are consistent with Deml et al.,^19^ who reported that the decision-making process is sometimes considered more important than the definitive selection of vaccines. Our participants perceived themselves as consultants for health topics and communicators on eye-level and they highly refrained from paternalistic medicine. Furthermore, their view on their occupational role differed from the orthodox image of a doctor, as the patient-centeredness and the empowerment of individual approaches were considered as the most important part of the medical service. A study by Reich^39^ that examined the beliefs of vaccination rejecting, highly educated, mostly full-time-mothers, showed similarities to our findings. These mothers believed in a maternal intuition that was more trustworthy than the knowledge of experts.

Some participants mentioned that their patients consulted them (i.e. a CAM practicing ‘Wahlarzt’), as they were disappointed by the way their biomedically oriented ‘Kassenarzt’ treated the vaccination topic. A study on reasons for CAM-use among patients with musculoskeletal conditions indicated the patients’ wish to increase control concerning health decisions and to close a perceived gap of care in terms of time and quality of the physician–patient relationship as important factors.^40^ The current situation in Austria with a fee-for-service scheme, a high workload among GPs and an average consultation time of 5 min per patient might counteract trust building physician–patient relationships that are associated with vaccine acceptance.^22^^,^^41–43^

### Distrust towards the medical system and the role of evidence-based-medicine

Similar to several other studies, we found distrust towards public health authorities and the pharmaceutical industry (e.g. References^37^,^38^). Peretti-Watel et al.^44^ described the importance of ‘trust issues’ in the emergence of VH. ‘Scientific scepticism has been extended to science itself and fuelled the disenchantment of science. […] Consequently, distrust towards science is no longer a sign of ignorance or even obscurantism, but is endorsed by highly educated individuals’. This statement may also be applied to some of our study participants and explain their perception towards vaccines in general.

Barry^45^ reported that the investigation of the effectiveness of CAM remedies/measures with the principles of evidence-based medicine usually plays an inferior role for CAM practitioners and that the emphasis for evaluating the efficacy of their treatment is focussed on their own experience and reports of colleges, friends and family members. In our study, the use of the term ‘evidence-based medicine’ was rather vague. Some study participants drafted a controversial image of vaccination that was in their point of view ‘evidence-based’. In turn, they blamed evidence-based medicine to be unreliable due to conflicts of interests as a result of financial support from pharmaceutical companies.

### Limitations

In the first place, our study aim was not to focus on VH among CAM practicing doctors. However, we experienced challenges to approach vaccine hesitant doctors who had no CAM background. The herewith presented sample includes 11 CAM-oriented ‘Wahlärzte’ and only one ‘Kassenarzt’ who practices orthodox medicine. We assume that our results may have been different if we had been able to recruit more doctors from orthodox medicine.

Our findings may also be valid for other high-income countries with a high demand for CAM. Nevertheless, they cannot be generalized, as participation in the study might have been a result of participants’ enhanced interest in the topic. Hence, one has to consider that this sample may represent a small, but vocal minority of CAM-oriented vaccine hesitant doctors.

## Conclusion

VH among doctors is a complex continuum of emotions, beliefs, narratives and values and is highly related to a perception of health and illness that is deviant to biomedical concepts. Our simultaneous examination of sentiments concerning the measles and its vaccine indicates that similar notions might be found for other childhood diseases and its corresponding vaccines (e.g. rubella, pertussis, mumps or varicella), as core elements in questioning the importance of the measles vaccine were the appraisal of the measles as an ‘ordinary childhood disease’ and the belief in positive effects arising from it. From the perspective of vaccine hesitant persons it would be illogic to prevent an infection, which is perceived as beneficial and only lethal if handled wrongly, by using a vaccine, which is regarded as harmful. Most participants were settled in their opinion and their distrust against the medical system enabled them to justify their position. Thus, it seems pointless to specifically address this vocal minority and provide them with public health information. Instead, approaches to mitigate VH among doctors should start at an earlier stage. Firstly, we suggest that information on vaccines and VH should be more firmly integrated into medical curricula. Secondly, appropriate communication techniques that address VH should be included in postgraduate education.

Furthermore, we suggest that ‘vaccination advice’ should be reimbursed by the Austrian social insurance in order to guarantee that information seeking patients receive science-based information in adequate quality and duration.

## Supplementary data

Supplementary data are available at *EURPUB* online.

## Supplementary Material

ckab174_Supplementary_Data
