# Supplementary material for: ‘I’m not an anti-vaxer!’—vaccine hesitancy among physicians: a qualitative study
Source: Eur J Public Health. 2021 Sep 28;31(6):1157–63. doi: 10.1093/eurpub/ckab174 (PMC8675240; doi:10.1093/eurpub/ckab174)
Supplement: ckab174_Supplementary_Data [file ckab174_Supplementary_Data.docx]

**Supplementary file – additional references**

41. Hoffmann K, Wojczewski S, George A, Schäfer WLA, Maier M. Stressed and overworked? A cross-sectional study of the working situation of urban and rural general practitioners in Austria in the framework of the QUALICOPC project. Croat Med J. 2015;56:366–74.

42. Redaèlli M, Wilm S, Simic D. Observatory Studies Series [Internet]. No. 40. Kringos DS, Boerma WGW, Hutchinson A, editor. Building primary care in a changing Europe: Case studies. Copenhagen: European Observatory on Health Systems and Policies; 2015. Available from: https://www.ncbi.nlm.nih.gov/books/NBK459015/

43. Kundi M, Obermeier P, Helfert S, Oubari H, Fitzinger S, Yun JA, et al. The impact of the parent-physician relationship on parental vaccine safety perceptions. Curr Drug Saf. 2015;10(1):16–22.

44. Peretti-Watel P, Larson HJ, Ward JK, Schulz WS, Verger P. Vaccine hesitancy: Clarifying a theoretical framework for an ambiguous notion. PLOS Curr Outbreaks. 2015;25(1):1–11.

45. Barry CA. The role of evidence in alternative medicine: Contrasting biomedical and anthropological approaches. Soc Sci Med. 2006;62(11):2646–57.
